# Muscle synergies evoked by microstimulation are preferentially encoded during behavior

**DOI:** 10.3389/fncom.2014.00020

**Published:** 2014-03-05

**Authors:** Simon A. Overduin, Andrea d'Avella, Jose M. Carmena, Emilio Bizzi

**Affiliations:** ^1^Department of Electrical Engineering and Computer Sciences, University of CaliforniaBerkeley, CA, USA; ^2^Laboratory of Neuromotor Physiology, Santa Lucia FoundationRome, Italy; ^3^Helen Wills Neuroscience Institute, University of CaliforniaBerkeley, CA, USA; ^4^UCB-UCSF Joint Graduate Group in Bioengineering, University of CaliforniaBerkeley, CA, USA; ^5^Department of Brain and Cognitive Sciences, McGovern Institute for Brain Research, Massachusetts Institute of TechnologyCambridge, MA, USA

**Keywords:** motor, movement, muscle, synergy, hand, macaque, grasping, cortex

## Abstract

Electrical microstimulation studies provide some of the most direct evidence for the neural representation of muscle synergies. These synergies, i.e., coordinated activations of groups of muscles, have been proposed as building blocks for the construction of motor behaviors by the nervous system. Intraspinal or intracortical microstimulation (ICMS) has been shown to evoke muscle patterns that can be resolved into a small set of synergies similar to those seen in natural behavior. However, questions remain about the validity of microstimulation as a probe of neural function, particularly given the relatively long trains of supratheshold stimuli used in these studies. Here, we examined whether muscle synergies evoked during ICMS in two rhesus macaques were similarly encoded by nearby motor cortical units during a purely voluntary behavior involving object reach, grasp, and carry movements. At each microstimulation site we identified the synergy most strongly *evoked* among those extracted from muscle patterns evoked over all microstimulation sites. For each cortical unit recorded at the same microstimulation site, we then identified the synergy most strongly *encoded* among those extracted from muscle patterns recorded during the voluntary behavior. We found that the synergy most strongly evoked at an ICMS site matched the synergy most strongly encoded by proximal units more often than expected by chance. These results suggest a common neural substrate for microstimulation-evoked motor responses and for the generation of muscle patterns during natural behaviors.

## Introduction

In numerous studies, motor primitives have been defined as “synergies” in which a group of muscles is simultaneously recruited, each with a specific balance of activation. These investigations have involved frog axial and hindlimb behaviors (Tresch et al., 1999), cat axial and hindlimb behaviors (Ting and Macpherson, 2005) and forelimb reaches (Yakovenko et al., 2010), rat forelimb reaches and grasps (Kargo and Nitz, 2003), monkey forelimb reaches and grasps (Brochier et al., 2004; Overduin et al., 2012), and human axial and hindlimb behaviors (Torres-Oviedo and Ting, 2007) and forelimb reaches, grasps and gestures (d'Avella et al., 2006; Klein Breteler et al., 2007; Berger et al., 2013). Analogous synergies have also been defined at the kinematic level (e.g., Santello et al., 1998; Gentner and Classen, 2006; Gentner et al., 2010).

Much of the direct evidence for synergistic muscle control by the central nervous system (CNS) comes from studies of the spinal cord. For instance, chemical intraspinal microstimulation in the frog evokes topographically-organized, low-dimensional electromyographic (EMG) activity patterns (Saltiel et al., 2001). In spinalized frogs, too, neurons including those in the intermediate zone of the spinal cord are better correlated with synergistic premotor drives than to the activity of individual muscles (Hart and Giszter, 2010). The spinal cord of primates contains premotor interneurons facilitating multiple muscles, including those intrinsic to the hand (Takei and Seki, 2010), and has been proposed as a substrate for synergies (Tresch et al., 1999; Cheung et al., 2009). Phasic activation of such units may be responsible for multi-muscular EMG bursts (Kargo and Giszter, 2008).

Synergies may be encoded at supraspinal as well as spinal levels, e.g., in the brainstem (Roh et al., 2011). At the level of the primary motor cortex (MI), in rodents learning a reaching task the firing rates of a minority of neurons are correlated with changes in the activation of synergies extracted from forelimb muscle EMG activity (Kargo and Nitz, 2003). Continuous neural control of synergistic muscle groups in primates is also circumstantially suggested by the ability to reconstruct forelimb EMG profiles as the weighted sum of an ensemble of neurons (Morrow and Miller, 2003; Schieber and Rivlis, 2007). MI neurons have muscle fields (defined by the strongest cell-EMG correlations) that appear to fall into relatively few, synergy-like clusters (Holdefer and Miller, 2002). Such cortical muscle fields may be “hard-wired,” changing their structure rarely, if ever (Kargo and Nitz, 2003). This may be particularly true of corticospinal and corticomotoneuronal cells, which facilitate small sets of muscles relatively directly (Fetz and Cheney, 1980; Bennett and Lemon, 1994). The latter cell population may define a “new MI” that affords muscular coordination unconstrained by synergies encoded in the spinal cord (Rathelot and Strick, 2009).

As with intraspinal microstimulation, application of relatively long trains of suprathreshold electrical current to individual sites in motor cortex can evoke complex movements. In cats (Ward, 1938), rats (Haiss and Schwarz, 2005; Ramanathan et al., 2006), prosimians (Stepniewska et al., 2005, 2011), and macaques (Graziano et al., 2002a, 2004b, 2005), suprathreshold microstimulation trains lasting several hundred milliseconds evoke complex multijoint forces that frequently drive the animal's body toward invariant postures. Shorter-train (<100 ms) intracortical microstimulation (ICMS), in contrast, typically evokes simpler twitch-like movements, often restricted to single joints (Graziano et al., 2002a; Stepniewska et al., 2011).

In the case of forelimb and hand areas of non-human primate motor cortex, ICMS has been shown to evoke behavioral fragments including reaching and defensive motions (Graziano et al., 2002a; Kaas et al., 2013). It has been suggested that motor cortical areas may be defined by a continuous map of endpoint postural space (Graziano et al., 2002b), e.g., divided according to the 3D regions around the monkey to which the forelimb is driven by ICMS (Graziano et al., 2002a), or to distinct behaviors like reaching and defending (Graziano et al., 2005). Whatever the nature of the topographical clustering of ethologically-relevant movements on the motor cortical surface, this organization appears to be reflected by distinct, interconnected regions in premotor and posterior parietal cortex (Stepniewska et al., 2005, 2011, 2013; Gharbawie et al., 2011).

Recently, we demonstrated that ICMS of monkey motor cortex elicited EMG patterns that could be decomposed into muscle synergies—ones similar to those seen in natural behavior (Overduin et al., 2012; for discussion see also Diedrichsen and Classen, 2012; Bizzi and Cheung, 2013; Santello et al., 2013). These EMG patterns co-occurred with movements of the hand toward a convergent posture. Like the apparent topographical organization of spinally-encoded synergistic limb movements (Tresch et al., 1999; Bizzi et al., 2000, 2002, 2008; Tresch et al., 2002), we also observed a non-uniform representation of each forelimb synergy on the cortical surface of macaques (Overduin et al., 2012). Much as microstimulation of multiple points in the spinal cord of frogs (Mussa-Ivaldi et al., 1994; Lemay et al., 2001) and rats (Tresch and Bizzi, 1999) evokes a linear summation of convergent forces and end posture, when ICMS is applied at multiple points in the motor cortex of anesthetized cats, the evoked EMG activity tends to sum linearly (Ethier et al., 2006). Together these results suggest a role of muscle synergies in simplifying (even to the point of linearizing) motor control.

Yet even if ICMS is able to evoke movements through combination of hard-wired muscle synergies, this does not imply that the synergies are *organized* intracortically. In humans, for instance, cortical stroke appears to spare some or most synergies (Cheung et al., 2009, 2012; Cruz and Dhaher, 2009), suggesting encoding at a subcortical locus. In the absence of descending signals from the brain, the spinal cord remains fully capable of generating complex behaviors and muscle activations (Pearson and Rossignol, 1991; Zimmermann et al., 2011), as well as microstimulation-evoked convergent forces (Giszter et al., 1993; Aoyagi et al., 2004).

Here, we examined motor cortical activity during voluntary behavior to see if it might play any role in controlling the activation (if not the structure) of muscle synergies. In particular, we tested a simple experimental hypothesis motivated by our earlier microstimulation work (Overduin et al., 2012), namely that cortical units should preferentially encode synergies similar to those evoked by ICMS at the same electrode. (Our null hypothesis, in contrast, was that whichever synergy was most strongly encoded by a given unit would bear only at-chance similarity to those evoked by ICMS near the unit.) If so, this would suggest that intracortical currents—whether endogenously generated by motor planning or exogenously introduced by microstimulation—may determine the degree to which downstream synergies are recruited.

## Materials and methods

### Subjects

Behavioral, muscular, and cortical data were collected from two rhesus macaques (*Macaca mulatta*): G1 (a 5.9-kg, 8-year-old female) and G2 (a 6.5-kg, 4-year-old male). Procedures were approved by the MIT Committee on Animal Care, and conformed to the National Institutes of Health *Guide for the Care and Use of Laboratory Animals*.

### Behavior

Subjects used their left hand to press a start button and then reach for, grasp, and carry objects between two wells spaced 20 cm apart. Reward (0.2–0.3 ml of apple juice or water) was given if the object was removed from the first well within 1.0 s, and released into the second well within another 1.0 s, where it had to remain for at least 0.1 s. Button press, reward dispensation, and data from two photosensors (E3T-SR12; Omron, Kyoto, Japan) mounted within each well were recorded together, allowing trials to be divided into reach and carry phases (Overduin et al., 2008). The 25 Delrin plastic objects (density 1.4 g/cm^3^) included 5 spheres of variable diameter (ranging from 1.6 to 3.6 cm), 5 cubes of variable width (1.5–3.6 cm), and 15 cylinders of which 5 each spanned one of three dimensions (height, 0.6–5.7 cm; uniform diameter, 1.3–3.8 cm; inner diameter, 0.6–3.2 cm). The number of objects presented in a given day, or spanned by any one single unit recorded in a given session, could be fewer than 25. The same object was presented enough times consecutively for the animal to be able to perform 10 successful trials, before another object was pseudorandomly selected. During recordings, subjects were head-restrained via an implanted cranial post.

### Sessions

Cortical recordings from monkey G1 comprised 7798 successful left-target-directed trials performed over 20 recording sessions spanning 45 days; those from G2 comprised 775 left-target-directed trials performed over 6 sessions spanning 6 days. The analysis presented here, however, was focused on the subset of these data for which ICMS experiments were performed on the same days as the cortical recordings (4485 trials over 13 sessions from G1 and 544 trials over 4 sessions from G2). Muscle recording was done in each of these sessions from G1 and G2, and also included trials (as well as other, interspersed sessions) when cortical recording was not done. The EMG data from which synergies were extracted (Overduin et al., 2008, 2012; Figure 3A) thus include trials recorded both with and without simultaneous neural data. In particular, of G1 and G2's 1000 left-target-directed trials used to construct trial-averaged EMG activity for synergy extraction, 482 and 462 trials overlapped with the 4485 and 544 trial subset we focus on here, respectively. The EMG data of the remaining 89% (4003/4485, G1) and 15% (82/544, G2) of trials are new to this report. None of the cortical data have previously been presented.

### Surgery

Cortical surgeries followed (G1) or occurred along with the first of (G2) the muscle electrode implantation surgeries (described in Overduin et al., 2008). These surgeries were performed under sterile conditions and general anesthesia (0.05 mg/kg atropine and 10 mg/kg ketamine injected intramuscularly, followed in G1 by 5 mg/kg sodium pentobarbital intravenously or in G2 by inhalation of 1–2% isoflurane with 2 L O_2_). Craniotomies were centered over right-hemisphere motor cortex. Custom stainless steel wells (G1: 28 mm wide, G2: 20 mm) were secured with bone screws and bone cement. The animals were given analgesics and systemic antibiotics following the surgeries. The dura was kept intact during surgery, and was subsequently treated with topical antibiotics and anti-inflammatories. Fresh connective tissue growth above the dura was further controlled by periodic (~ 1× weekly) mechanical scraping, done under light anesthesia through the weeks of recording.

### Muscles

EMG recordings were made via 15 (G1) or 19 (G2) electrodes chronically implanted in muscles of the left forelimb. Proximal muscles acting on the shoulder and elbow included Del (deltoideus), Pec (pectoralis major), TriU and TriR (triceps brachii, ulnar and radial short heads), Bic (biceps brachii longus), and BR (brachioradialis). Wrist and extrinsic hand extensors included AbPL (abductor pollicis longus), ECRB (extensor carpi radialis brevis), EDC (extensor digitorum communis), ED23 (extensor digiti secundi and tertii proprius), ED45 (extensor digiti quarti and quinti proprius), and ECU (extensor carpi ulnaris). Wrist and extrinsic hand flexors included FCR (flexor carpi radialis), FDS (flexor digitorum superficialis), FDPU and FDPR (flexor digitorum profundus, ulnar and radial), and FCU (flexor carpi ulnaris). Intrinsic hand muscles included AbPB (abductor pollicis brevis), AdP (adductor pollicis), OpP (opponens pollicis), F5B (flexor digiti quinti brevis manus), and Op5 (opponens digiti quinti manus). EMG data were recorded on a trial-by-trial basis (between button-press and reward events). These data were bandpass-filtered (between 10 and 1000 Hz), notch filtered (60 Hz), and differentially amplified (5000×) by a programmable signal conditioner (CyberAmp 380; Molecular Devices, Sunnyvale, CA) under software control (CyberControl; Molecular Devices). Data were digitized (2 kHz) via a data acquisition board (NI PCI-6035E; National Instruments, Austin, TX) under custom software control (LabVIEW; National Instruments). EMG channel subselection following cross-talk analysis is described in Overduin et al. (2008).

### Cortex

**Figure 1:**
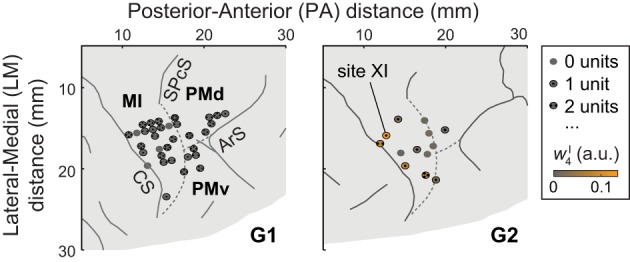
**A portion of each monkey's right hemisphere as viewed from above**. Solid lines represent sulci (CS, central sulcus; SPcS, superior precentral sulcus; ArS, arcuate sulcus); dashed lines show estimated inter-areal borders separating MI, PMd, and PMv. The circles show stimulation and recording sites, with dots indicating the number of units (if any) recorded at the site. For G2, ICMS sites are colored according to the degree to which the evoked muscle EMG activity was similar to (i.e., reconstructed by) synergy **ν**^I^_4_. (Specifically, the color scale indicates the mean amplitude coefficient *w*^I^_4_ used in reconstructing the ICMS-evoked EMG vectors at a site with synergy **ν**^I^_4_, averaged over ICMS trains.) Of G2's ICMS sites, this synergy was most strongly evoked at site XI, in MI.

**Figure 2:**
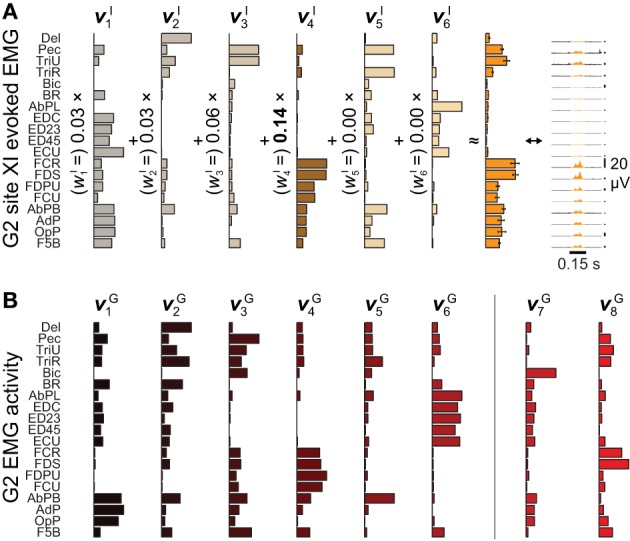
**Microstimulation-evoked muscle activity and reconstruction by synergies. (A)** The EMG activity evoked during the first 0.15 s of ICMS at site XI from Figure 1, shown both in gold-colored profile (*far right*) and as the integrated mean ± SE of this activity over stimulation trains (*second from right*). (The vertical 20-μV scale bars to the right of the EMG profiles give the relative level of activation in each channel.) The mean activity could be reconstructed by six ICMS-evoked synergies **ν**^I^_*n*_ (*left*), with the largest weighting given to **ν**^I^_4_. **(B)** Of eight muscle synergies **ν**^G^_*n*_ derived from an object grasping behavior, synergies **ν**^G^_1_−**ν**^G^_6_ could be matched in structure to ICMS-evoked synergies **ν**^I^_1_−**ν**^I^_6_.

Single units were recorded from dorsal premotor cortex (PMd), ventral premotor cortex (PMv), and MI (Figure 1). Areas were identified using magnetic resonance imaging data and sensorimotor mapping including ICMS. MRIs were collected 2 years after (G1) or 4 months before (G2) the recordings presented here. At the beginning of recording sessions, unit somatosensory (proprioceptive and cutaneous) response fields were estimated by passively moving the monkey's limbs and by stimulating its skin. Both in early exploratory sessions and at the end of each recording session, ICMS was applied via tungsten microelectrodes (FHC, Bowdoin, Maine; 0.3–3-MΩ impedance, 250-μ m shaft diameter tapered to a 3-μ m-wide tip). Up to 10 such electrodes were acutely introduced into the brain in each session using a manual microdrive (30-μ m depth resolution). The microdrive was mounted on a grid that was secured to the recording well and that constrained the interelectrode spacing to 1 mm. The ICMS-evoked movements and stimulation thresholds were used to identify the portion of cortex sampled by the electrodes. Stimulation parameters used for this mapping included 2 × 0.2-ms pulse duration (cathodal-leading), 10–150-μ A current, 330-Hz pulse frequency, and 0.05-s train duration. Modified ICMS parameters were used to evoke longer-lasting movements at the end of some sessions (Figure 2A): 8–100-μ A current, 200-Hz pulse frequency, and 0.15–0.5-s train duration (as in Overduin et al., 2012). We consider only the first seven ICMS trains delivered at each site, this being the minimal number applied across the 33 (G1) or 13 (G2) sites (including 32 in MI, 9 in PMd, and 5 in PMv; Overduin et al., 2012).

### Units

Extracellular, intracortical voltages were recorded through the same electrodes used for ICMS. During recordings, no attempt was made to record from units within particular cortical layers or in sulcal sites, and the laminar location of recorded units was generally unknown given limited depth resolution and dimpling of the cortex upon penetration. Instead, we selected for recording the first stably-firing and well-discriminated unit(s) (if any) discovered along a given electrode track, as revealed by audio and oscilloscope monitoring of the recorded voltage. We rejected all cortical sites wherein somatosensory stimulation or ICMS had indicated response fields extending beyond the left forelimb (e.g., to the face or leg). We did not require the remaining sites to demonstrate sensorimotor responses, only that they be within topographical regions convexly bounded by sites that did. No other criteria were applied to units; i.e., we treated all as potentially task-related. We only consider units recorded at ICMS electrode sites (33 sites in G1, 13 in G2; Overduin et al., 2012); these comprise 94 units (83 in G1, 11 in G2). Signals were preamplified at unity gain by a headstage located ~5 cm from the electrodes, and then passed to an amplifier for amplification (10,000×) and bandpass-filtering (600–6000 Hz, 2nd-order filter with roll-off on both ends) before digitization (Neuralynx, Inc., Tucson, Arizona). Spikes were identified online when electrode voltages exceeded a manually-set threshold, and 1.1-ms waveforms (including the threshold-crossing moment at 0.26 ms into the waveform), sampled at 30 kHz, were stored to disk. Spike times and other event times were recorded together for later synchronization of behavioral, EMG, and neural data. Offline, single units were identified based on spike waveform features and interspike intervals (ISIs), using manual clustering with MClust (MClust-3.4, A. D. Redish et al.) and custom routines written in MATLAB (MathWorks, Natick, Massachusetts), for this and the following analysis. Particular care was taken to ensure that waveform features and firing rates remained relatively constant over the recording span of each accepted unit. The 94 units had mean firing rates between 1.0 and 58.0 Hz, and signal-to-noise ratios between 2.4 and 24.3 (deCharms et al., 2009), with 1% of ISIs <1 ms.

### Preprocessing

**Figure 3:**
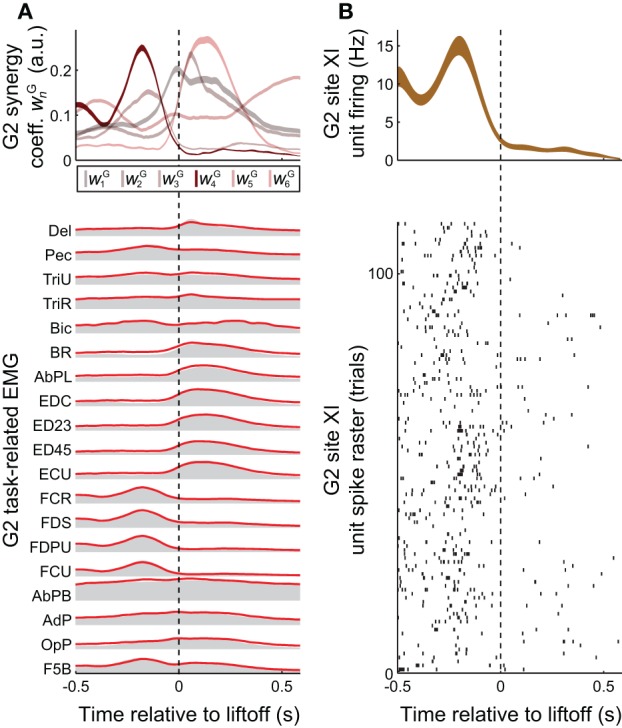
**Task-related muscle activity and reconstruction by synergies, vs. cortical activity. (A)** EMG activity in the grasping task (gray envelopes at *bottom*) could be decomposed as a summation of grasping-related synergies **ν**^G^_*n*_ (*red* lines at *bottom*), each weighted by the coefficients *w*^G^_*n*_ (*top*). EMG activity and synergy coefficients are shown as mean (*bottom*) and mean ± SE (*top*), over all 25 objects and 733 trials spanned by this monkey's EMG dataset. **(B)** The task-related firing rate of a unit recorded at monkey G2' site XI, shown both as a spike raster (*bottom*) and as the binned activity mean ± SE (over the 22 objects and 113 trials spanned by this unit, *top*). Of all the task-related synergies **ν**^G^_1_−**ν**^G^_6_ used in reconstructing EMG activity unit's activity (**A**, *top*), the one corresponding to **ν**^I^_4_ (i.e., **ν**^G^_4_) had an activation profile most similar to the unit's activity (**B**, *top*).

**Figure 4:**
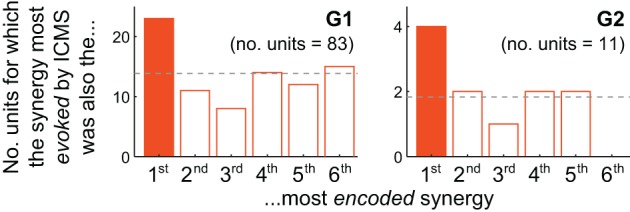
**Motor cortical units are biased toward encoding synergies evoked by nearby microstimulation**. For both G1 and G2, the muscle synergy most strongly evoked by ICMS was also the synergy most likely to be best-correlated with the firing of cortical units at the same electrode. The unit depicted in Figure 3B, for instance, contributes to G2's solid-colored bar.

After preprocessing as described in Overduin et al. (2008), grasping-related EMG data were integrated over 9-ms (G1) or 11-ms (G2) bins, and normalized to each channel's maximum integrated EMG over object conditions. The EMG data of 40 trials within each of the 25 object conditions were aligned on the time of object removal from the first well, cropped to a 100-sample window around this event [G1:(−0.35 : +0.55) s, G2:(−0.5: +0.6) s], and then averaged over trials within each object condition. (The different window and integration times for the two animals were chosen based on their different movement latencies; Overduin et al., 2008.) ICMS-evoked EMG data were integrated over a (+0.025: +0.150) s window relative to ICMS onset for each of 7 trains applied at each stimulation site, and normalized by the same factors as the grasping-related EMG data (Overduin et al., 2012). With regards to the neural data, mean firing rates were computed on a within-object basis, using all trials fully spanned by a unit. Trials were time-aligned on the time of object removal from the origin well (as for the grasping-related EMG data). Subsequent analysis was restricted to a (−0.4: +0.5) s (G1) or (−0.55: +0.55) s (G2) window around this event, i.e., to the same windows as for the grasping-related EMG data minus a fixed 50-ms delay (Morrow and Miller, 2003; Schieber and Rivlis, 2007; Stark et al., 2007). Within this window, each unit's spikes were summed within 9- or 11-ms bins (again, for consistency with the grasping-related EMG data binning). The units' mean firing rate profiles within each object condition were then smoothed by convolution with a 50-ms Gaussian kernel. (This smoothing, evident in Figure 3B, did not have a qualitative effect on the summary results of Figure 4.) All preprocessing was done in MATLAB.

### Synergies

Non-negative matrix factorization (NNMF) was used to identify a set of synchronous synergies underlying each monkey's muscle patterns (Lee and Seung, 1999; Tresch et al., 1999). There were two sets of muscle patterns to consider: ICMS-evoked EMG data ***I*** (including the data evoked at the sample site in Figure 2A, *right*), and grasping-related EMG data *G* (shown averaged over object conditions in Figure 3A, *bottom*). Matrix ***I***(*e, s, l*) pools together the activity in *E* EMG channels evoked by each of *S* stimulation trains delivered at each of *L* ICMS locations (Overduin et al., 2012). ***I*** thus has dimensionality 15 × 7 × 33 (G1) or 19 × 7 × 13 (G2). Matrix ***G***(*e, t, o*) specifies trial-averaged EMG activity over the same *E* channels, but across each of *T* time points in each of the *O* object conditions. *G* thus has dimensionality 15 × 100 × 50 (G1) or 19 × 100 × 50 (G2). Data ***I*** and ***G*** were independently reconstructed as combinations of *n* = 1, …, *N*^I^ or *n* = 1, …, *N*^G^ synergies, respectively, where both *N*^I^ and *N*^G^ were evaluated up to the number of EMG channels *E*, i.e., 15 (G1) or 19 (G2). Each ICMS-evoked or grasp-related synergy **ν**^I^_*n*_(*e*) or **ν**^G^_*n*_(*e*) is a vector of length *E* capturing a unique balance of activation across the EMG channels. In reconstructions, each synergy was multiplied by a scalar, non-negative weighting coefficient *w*^I^_*n*_(*s, l*) or *w*^G^_*n*_(*t, o*). These coefficients could vary both within conditions (i.e., over stimulation trains *s* or time samples *t*) and across conditions (i.e., over ICMS locations *l* or objects *o*). The reconstructions can be expressed as:

(1) $$I(e,\;s\;,l)=\sum_{n\;=\;1}^{N^{I}}w_{n}^{I}(s,\;l)\cdot \nu_{n}^{I}(e)$$

(2) $$G(e,\;t,\;o)=\sum_{n\;=\;1}^{N^{G}}w_{n}^{G}(t,\;o)\cdot \nu_{n}^{G}(e)$$

For a given *N*^I^ or *N*^G^, the algorithms iteratively updated the structures **ν**^I^_*n*_ or **ν**^G^_*n*_ and coefficients *w*^I^_*n*_ and *w*^G^_*n*_ until the total reconstruction error, *R*^2^, increased by less than 0.001 over 10 iterations. The algorithms were repeated five times for each extraction; the set of synergies with the highest EMG variation explained was selected for further analysis. Dimensionalities (*N*^I^ and *N*^G^) were chosen by applying a threshold of *R*^2^ = 95% (Overduin et al., 2012).

### Matching

Synergies **ν**^I^_*n*_ were compared and matched to synergies **ν**^G^_*n*_ using a greedy search procedure (Tresch et al., 1999; Overduin et al., 2012). For all *N*^I^ × *N*^G^ possible pairs of ICMS-evoked and grasping-related synergies, we first computed dot products (e.g., 6 × 8 = 48 dot products, for G2. The pair of ICMS-evoked vs. grasping-related synergies with the highest dot product was defined as the best-matching pair. The pair with the highest dot product among the remaining (*N*^I^ − 1) × (*N*^G^ − 1) pairs (e.g., 5 × 7 = 35 pairs, for G2) defined the second-best match. This process was repeated until all synergies in one set had been paired (e.g., over min(6,8) = 6 times for G2). We then used Monte Carlo simulation to assess the significance of each match. We repeated the greedy search algorithm 10,000 times for each monkey, after first randomly shuffling EMG channel identity each time. (For G2, for instance, this involved finding 10,000 × 48 = 480,000 dot products.) We then compared the highest (best-matching) dot product between *actual* ICMS-evoked and grasping-related synergies with the distribution of highest dot products from the 10,000 comparisons of *shuffled* synergies. If the former value exceeded the 95th percentile of the distribution of latter values, we took the match as significant at *p* < 0.05. We then repeated this comparison for the second-best actual synergy pair vs. the distribution of second-best shuffled pairs, etc.

### Analysis

In reconstructing the ICMS-evoked EMG vectors at a given site, the synergy **ν**^I^_*n*_ most “evoked” at the site was the one with the largest weighting coefficient *w*^I^_*n*_, averaged over ICMS trains (Figure 2A, showing results for site XI in Figure 1, *right*). To determine which synergy was instead most “encoded” by a unit, the unit's firing rate profile was correlated against the task-related synergy scaling profiles *w*^G^_*n*_, and the largest positive correlation was identified. Results were insensitive to the use of linear (Pearson) or rank (Kendall or Spearman) correlation. To determine which task-related synergy was most evoked at a recording site, the site's ICMS-evoked EMG patterns were decomposed into combinations of synergies, which in turn were matched to task-related synergies (as described above). Over units, we counted the frequency at which the most-encoded synergy was the same as the most-evoked synergy at the electrode, defined as above. The chance frequency of such matches was 1/6 = 17%, as the analysis considered the 6 muscle synergies both evoked by ICMS (in the case of G2, Figure 2A, *left*) and observed in the task data (Figure 2B). Results of this χ^2^ test were evaluated for significance at a *p* < 0.05 threshold.

## Results

In a recent study (Overduin et al., 2012), we examined ICMS-evoked movements and muscle activity in two rhesus macaques (“G1” and “G2”). EMG data were recorded from 15–19 electrodes chronically implanted in muscles of the shoulder, arm, and hand. As we reported (Overduin et al., 2012), ICMS within MI and dorsal and ventral premotor (PMd and PMd) cortex (Figure 1) appeared to drive the forelimb toward an invariant, site-specific posture, and at the same time to replace voluntary muscle activity with an invariant, site-specific tonic EMG pattern (as in Griffin et al., 2011).

After aggregating ICMS-evoked EMG data over multiple stimulation sites, these pooled data could be decomposed into combinations of a reduced set synchronous synergies using NNMF (Lee and Seung, 1999; Tresch et al., 1999). Each of the ICMS-derived synergies **ν**^I^_*n*_ *n* = 1,…,*N*^I^) captures a pattern of synchronous firing over muscles. We found that *N*^I^ = 6 (G2) or 7 (G1) synergies were sufficient to reconstruct ≥ 95% of the variability in the ICMS-evoked EMG data (Overduin et al., 2012). Such data reconstruction is exemplified in Figure 2A for G2's ICMS site XI, in MI. The figure depicts the ICMS-evoked activity at this site in the form of: the time-varying, trial-averaged EMG signal (*far right*); an ICMS-evoked EMG vector integrating this per-ICMS activity over time (*middle*); and as a vector sum of ICMS-derived synergies, each scaled by the weighting coefficient *w*^I^_*n*_ (*left*). For this site, the ICMS-evoked EMG activity was dominated by synergy **ν**^I^_4_, as shown.

We also studied a manual behavior performed by these subjects (Overduin et al., 2008, 2012), in which they had to reach for an object presented in a well, grasp it, and then carry it to the opposing well. To elicit a variety of hand postures and forces, the objects included 25 spheres, cubes, and cylinders of different dimension. Pooled over multiple days, the EMG data could be decomposed into combinations of grasping-related synergies **ν**^G^_*n*_ (*n* = 1,…,*N*^G^). We found that *N*^G^ = 8 (G2) or 10 (G1) synergies were sufficient to reconstruct ≥ 95% of the variability in the grasping-related EMG data (Overduin et al., 2012). For each monkey, 6 of these synergies could be matched uniquely to one of the ICMS-derived synergies (Figures 2A vs. 2B) using a greedy search procedure. Figures 2B, 3A illustrate the synergies found for one animal and their reconstruction of average EMG activity.

Synergies could provide the animal with a mechanism to continuously and efficiently control its muscles, by specifying a task-specific amplitude coefficient time course *w*^G^_*n*_ for each of the synergies (Figure 3A *top* vs. *bottom*). The involvement of motor cortex in this control is circumstantially suggested by the correspondence in synergy structure whether these are derived from ICMS-evoked or voluntarily-generated EMG data (Figures 2A vs. 2B). Here, we sought more direct evidence for the role of motor cortex. In particular, we looked for evidence that the muscle synergies evoked by ICMS (and matched to those generated voluntarily) were also encoded by single motor cortical units near the ICMS electrodes.

A positive example of such a correspondence is shown in Figure 3A (*top*) and Figure 3B (*top*), for monkey G2. The MI unit shown in Figure 3B is at site XI, the location of which is highlighted in Figure 1 (*right*), and the ICMS-evoked EMG activity of which is shown in Figure 2A. The single unit recorded at this site exhibited a strong burst of activity just prior to the time when the object was retrieved from its origin well, and was largely quiet during the following carry and release movements (Figure 3B). (These modulations were captured in the unit's firing rate, derived from spike counts binned at 50 ms and smoothed with a 50-ms Gaussian kernel.) Of all the grasping-related synergies **ν**^G^_1_ − **ν**^G^_6_, the one whose activation profile was most positively correlated with that of the unit was **ν**^G^_4_ (Figure 3A, *top*), which appeared to capture the coactivation of forearm flexor muscles (Figure 2B). And of all the ICMS-evoked synergies **ν**^I^_1_ − **ν**^I^_6_, it was the matching synergy **ν**^I^_4_ that was most dominant in reconstruction of the evoked EMG activity.

How consistently did the muscle synergy most encoded by a unit match the synergy most evoked by ICMS at the same site (as in the foregoing example)? For this analysis we simply counted the number of MI, PMd and PMv units for which the grasping-related synergy most strongly encoded by the unit matched the primary muscle synergy evoked by ICMS at the electrode. (This analysis is restricted to those ICMS sites at which units were also recorded; these are highlighted in Figure 1.) As shown in Figure 4, the actual frequencies were significantly higher than the 1/6 = 17% chance frequency [26 of 94 units, or 28%; χ ^2^_(1)_ = 6.82, *p* < 0.01], as was true for G1 alone [22/83 = 27% of units; χ ^2^_(1)_ = 4.82, *p* < 0.05] and supported by a trend among G2's smaller population of units [4/11 = 36% of units; χ^2^_(1)_ = 2.56, *p* = 0.07]. (Note that the “1st most encoded synergy” in this plot is not necessarily **ν**^G^_1_, but instead whichever synergy **ν**^G^_*n*_ was most strongly correlated with the unit's firing profile, e.g., **ν**^G^_4_ for the unit shown in Figure 3B. Similarly, the “most evoked synergy” is whichever synergy **ν**^I^_*i*_ was dominant in reconstruction of the EMG activity evoked at the unit's electrode. In general, **ν**^I^_3_, **ν**^I^_4_, and **ν**^I^_5_ appeared to be most commonly evoked by ICMS, being the synergies “evoked” at 18/83, 28/83, and 20/83 of monkey G1's units, respectively, and by 3/11, 3/11, and 4/11 of G2's units.)

While the qualitative pattern for G2 followed very closely that of G1 (Figure 4), it likely failed to reach significance due to insufficient sampling of units (11 units, vs. 83 for G1). Also, these trends were weakened by inclusion of PMv units. Considering *only* MI and PMd units, the fraction of cases in which the grasping-related synergy most strongly encoded by a unit matched the synergy most strongly evoked by proximal ICMS was significantly higher than 1/6 in G2 [4/10 = 40% of units; χ ^2^_(1)_ = 3.27, *p* < 0.05] as well as G1 [20/73 = 27% of units; χ ^2^_(1)_ = 5.04, *p* < 0.05], and in both animals combined [24/83 = 29%; χ ^2^_(1)_ = 7.47, *p* < 0.01].

While the synergies **ν**^I^_*i*_ extracted from ICMS-evoked EMG activity did cluster non-uniformly on the cortical surface (Overduin et al., 2012), we observed no tendency of the subset of sites at which similar synergies were both encoded and evoked to be topographically grouped on the cortical surface.

## Discussion

Our results suggest that direct stimulation of patches of motor cortex generates synergistic combinations of muscle activity that are weighted toward synergies encoded at the stimulating electrode. A common motor cortical substrate appears to be activated both by endogenous currents during voluntary behaviors and by exogenous currents introduced by electrical microstimulation. In either case, these currents appear to modulate the amplitude, not structure, of a shared set of downstream muscle synergies. Our earlier work also suggests that each synergy (and the posture reached through its tonic activation) may be represented by a non-uniform map over the motor cortical surface (Overduin et al., 2012).

It may be contested that electrical currents injected into the CNS via microstimulation elicit non-natural patterns of neural activity (even if the downstream muscle activity is resolvable into well-organized movements and natural muscle synergies). The biological basis of ICMS, and even the extent of cortex activated, remain poorly understood (Butovas and Schwarz, 2003; Tolias et al., 2005), despite recent studies using single-cell recording, behavioral methods, functional magnetic resonance imaging (Tehovnik et al., 2006), and two-photon calcium optical imaging (Histed et al., 2009). Moreover, these investigations of ICMS have largely been limited to more conventional, short-train and/or subthreshold ICMS rather than the form used here. In applying ICMS in motor cortex, researchers have traditionally used short train durations (typically 25–70 ms) and sub- or per-threshold currents (typically 10–60 μ A; e.g., Asanuma and Rosén, 1972; Sato and Tanji, 1989; Donoghue et al., 1992) in order to map the overt response of cell populations including corticomotoneuronal cells. Such studies neither sought nor reported convergent movement responses of the sort found here and by others (Graziano et al., 2002a,b), and have naturally aroused some controversy (Strick, 2002).

Together with Overduin et al. (2012), our findings indicate that movements, muscle patterns, and cortical activations evoked by relatively long-train ICMS can be related to those observed in natural behavior. Indeed, it can be argued that a physiologically realistic model of motor activation *requires* ICMS with relatively long trains, as well as suprathreshold currents and intermediate pulse frequencies (optimally 80–140 μ A and 80–140 Hz, in the case of primate MI forelimb-area ICMS; Van Acker et al., 2013). Graziano and coworkers (Graziano et al., 2002a) have emphasized that ~500-ms stimulation trains approximate the time scale of natural movements like primate reach and grasp (Georgopoulos et al., 1986; Reina et al., 2001). Even in spinalized amphibians, in whom convergent force patterns were first observed (Mussa-Ivaldi et al., 1990; Giszter et al., 1993; Loeb et al., 1993), these movements were evoked by similarly long-train (typically 300-ms) intraspinal microstimulation. Researchers using ICMS to study oculomotor and somatosensory systems have also used relatively long trains. For instance, 400-ms trains were applied to the arcuate sulcus to replicate the time scale of typical regular head movements (Freedman et al., 1996), and 500-ms trains were applied to primary somatosensory cortex to mimic a tactile stimulus (Romo et al., 1998). While trains shorter than ~500 ms elicit truncated movements when delivered to primate MI (Graziano et al., 2002a; Van Acker et al., 2013), in Overduin et al. (2012) we show that even 150 ms of ICMS yields convergent movements with a predictable equilibrium point and synergistic muscle activation.

For the majority of units we sampled, firing rate profiles were more correlated with muscle synergies *other* than the most-evoked synergy (Figure 4, non-shaded bars). There are many ways to account for these other units. For example, units may encode other continuously-controlled quantities like the position, velocity, or acceleration of extrinsic effectors or intrinsic joints, or the forces underlying these kinematics, or the muscle contractions determining these dynamics (Carmena et al., 2003; Morrow and Miller, 2003; Schieber and Rivlis, 2007; Stark et al., 2007; Velliste et al., 2008; Ganguly and Carmena, 2009; Hochberg et al., 2012; Collinger et al., 2013). Motor cortical encoding of purely extrinsic variables may be unlikely based both on theoretical grounds (Todorov, 2000; Paninski et al., 2004) and on empirical evidence that neurons' intrinsic muscle fields are more stable than their tuning to extrinsic dimensions like hand direction (Morrow et al., 2007), with such tuning observed to fluctuate even during stable within-session behavior (Carmena et al., 2005; Rokni et al., 2007; cf. Chestek et al., 2007). However, the purpose of this report was not to determine whether motor cortical units encode muscle activity, synergy coefficients, or any other motor variable. Instead, we simply sought to test whether the units were more likely than chance to encode those synergies evoked by ICMS at the same electrode.

Another way to account for units which appeared to encode different synergies than those evoked by nearby ICMS is to recognize that the number of units affected by ICMS current far exceeds the handful sampled at an electrode. With the stimulation currents needed to elicit complex movements (≤100 μ A in this report), cortex is no doubt activated far outside the 100-μ m radius assumed for ~10 μ A stimulation (Ranck, 1981). Such suprathreshold trains are likely to recruit other cells beyond those immediately next to the stimulating electrode through synaptic connections (Histed et al., 2009), and indeed may be required to transsynaptically activate non-direct connections between motor cortex and the spinal cord (Strick, 2002).

Another possibility to be explored in future work is that populations of units may specify not only continuous variables like synergy amplitude, but also sequential recruitment of muscles (e.g., via synergies) in the form of discrete “motor programs” (Keele, 1968; Polit and Bizzi, 1978, 1979; Georgopoulos et al., 1983). Elsewhere these programs have been referred to as “time-varying synergies,” in distinction from the “synchronous synergies” discussed in the present report (Kargo and Nitz, 2003), and have been extracted from muscle data using a modified form of NNMF (d'Avella et al., 2003, 2006; Overduin et al., 2008). A dynamical systems approach may also be appropriate for capturing time-varying patterns within population-level neural and muscular data (Churchland et al., 2012).

Besides studies of voluntary neural and muscular dynamics, microstimulation studies are also broadly consistent with the idea of discrete movement encoding. Intraspinal microstimulation of sufficient duration generates forces that tend to drive a limb to particular postures or through sequences of postures, in frogs (Mussa-Ivaldi et al., 1990; Giszter et al., 1993; Kargo and Giszter, 2000), cats (Lemay and Grill, 2004) and rats (Tresch and Bizzi, 1999). Sufficiently-long ICMS trains applied to mammalian motor cortex (Ward, 1938; Graziano et al., 2002a, 2004a, 2005; Stepniewska et al., 2005, 2011; Ramanathan et al., 2006) can evoke complex multijoint forelimb behaviors with multiple phases (such as reach, grasp, then retraction) and invariant endpoints. The stimulation-evoked convergent forces (Giszter et al., 1993; Kargo and Giszter, 2000), invariant endpoints (Graziano et al., 2004a; Graziano and Aflalo, 2007), and bell-shaped speed profiles (Graziano et al., 2005) all tend to overlap with motions and postures found in subjects' natural behavior. Together with the present results, these studies indicate that microstimulation-evoked movements are not artifactual, and indeed can provide insights into natural movement planning.

### Conflict of interest statement

The authors declare that the research was conducted in the absence of any commercial or financial relationships that could be construed as a potential conflict of interest.
